# Influence of Dietary Cobalt on Fiber Digestibility and Serum Cobalt and Cobalamin Concentrations in Horses

**DOI:** 10.3390/ani14243595

**Published:** 2024-12-12

**Authors:** Rebecca Ashlee LeCompte Lazić, Brian D. Nielsen, Cara I. Robison, Harold C. Schott, Thomas H. Herdt, Connie K. Larson

**Affiliations:** 1 Department of Animal Science, 474 S. Shaw Lane, Michigan State University, East Lansing, MI 48824, USA; ashleelazic@gmail.com (R.A.L.L.); oconn107@msu.edu (C.I.R.); 2 Department of Large Animal Clinical Sciences, 784 Wilson Road, Michigan State University, East Lansing, MI 48824, USA; schott@msu.edu (H.C.S.II); herdt@msu.edu (T.H.H.); 3 Zinpro Corporation, Eden Prairie, MN 55344, USA; clarson@zinpro.com

**Keywords:** cobalt, equine, horse, fiber, cobalamin, folate, digestibility

## Abstract

The 2007 Horse NRC reduced cobalt (Co) requirements from 0.1 ppm to 0.05 ppm in dietary dry matter, though preliminary research suggested increasing dietary-Co enhanced fiber digestion in horses. This study aimed to evaluate fiber digestion with varying dietary Co amounts, as well as to compare serum Co concentrations with dietary Co content. Four geldings (mean body weight [BW] 503 ± 37 kg) were studied in a 4 × 4 Latin square design consisting of four 5-week periods, each comprising a 2-week washout phase followed by a 3-week treatment phase. Supplementing 0.0, 5.6, 16.8, or 28.0 mg of Co daily resulted in total Co dietary concentrations of 0.06, 0.7, 2.0, and 3.0 ppm, respectively. Feces and urine were collected to evaluate fiber digestibility and Co balance. Co supplementation did not affect neutral detergent fiber, acid detergent fiber, or lignin digestibility. Co balance was negative for the control diet but positive for the other three treatments (*p* = 0.04). Serum cobalamin concentrations slightly decreased (6%) with increasing dietary Co intake (*p* = 0.003), while folate concentrations were unaffected. Serum Co concentration differed (*p* < 0.01) with a mean of 4.7 μg/mL for the high treatment and 0.8 μg/mL for the control. Results suggest that serum Co concentrations above regulatory limits are likely due not to higher levels of Co in fortified feeds, but rather to parenteral administration of Co solutions.

## Simple Summary

There has been limited investigation of the cobalt (Co) requirements of horses. The 2007 Horse NRC established a requirement of 0.05 ppm in dietary dry matter. However, unpublished work has suggested that higher dietary Co could increase fiber digestion in horses. This study was designed to evaluate that observation and compare varying dietary Co amounts with serum Co concentrations, particularly as the horse racing industry faces concerns with elevated Co concentrations being associated with both potential performance enhancement and welfare concerns. While increasing dietary Co did not result in increased fiber digestion, this study showed that Co fed at 60 times the 2007 NRC requirement produced a mean serum Co concentration less than 20% of the upper limit accepted by racing jurisdictions, and well below the concentrations detected in post-race samples collected from horses suspected of parenteral Co administration. This study suggests that serum Co concentrations above regulatory limits are likely due not to higher levels of Co in fortified feeds, but rather to parenteral administration of Co solutions.

## 1. Introduction

Cobalt (Co) is necessary for microbial synthesis of vitamin B_12_ (cobalamin) in the horse [1]. Vitamin B_12_ serves as a growth factor for many microorganisms in the gastrointestinal (GI) tract and is an essential co-factor for gluconeogenesis in the host animal [2]. Along with its structural role in vitamin B_12_ synthesis, Co has also been suggested to increase fiber digestion in herbivores by acting as a divalent cation [3]. Non-starch polysaccharides and lignin, more commonly known as dietary fiber (DF), are main components of herbivore feedstuffs. The complex structure of these fibers renders them resistant to enzymatic digestion within the digestive system of mammals such as the horse [4]. To be able to digest DF, herbivorous animals have a symbiotic relationship with microflora within the GI tract that can ferment and degrade fiber. In foregut fermenters, breakdown of DF occurs in the rumen, producing volatile fatty acids (VFAs) that can be absorbed as an energy source.

In contrast, in hindgut fermenters including the horse, DF remains largely intact during passage through the small intestine [5]. While located throughout the gastrointestinal tract, the large microbial population in the hindgut ferment DF into VFAs in these species. There is limited research on a potential role of dietary Co content in fiber digestion in the horse. Fehlberg et al. explored the effects of Co on fiber digestion in an in vitro model using equine cecal fluid, and reported that increasing amounts of Co had limited impact on digestion of DF [6]. However, there has been no investigation of dietary Co on in vivo DF digestion in horses.

Cobalt has traditionally been used to treat anemia, because cobalamin (commonly known as vitamin B_12_) is essential for hematopoiesis and vitamin B_12_ deficiency can lead to pernicious anemia. Currently, pernicious anemia is treated by both oral and parenteral administration of vitamin B_12_, rather than Co supplementation, because of adverse effects of Co on thyroid gland function and cardiotoxicity with prolonged dietary supplementation [7]. Cobalt activates the hypoxia-inducible factor (HIF) pathway, which promotes glycolytic enzyme activity in response to decreased oxygen availability [8,9]. 

Over the past couple of decades, Co supplementation as an ergogenic aid has increased in human athletes. Cobalt-associated increased HIF expression can result in an increase in erythropoietin (EPO) and, subsequently, erythrocyte production to a similar degree as that produced by administration of recombinant human EPO [10]. Co supplementation to enhance equine performance had not been apparent until about 10 years ago. A string of race-related deaths at Hollywood Park racetrack in California had officials speculating that Co could be a factor in the sudden death of seven Thoroughbred racehorses over a 16-month period; all horses belonged to the same trainer and were under the medical care of the same veterinarian [11]. A mineral that had received little attention in the equine community became of critical interest in equine welfare [12]. 

The intent of this study was to evaluate the effects of varying amounts of dietary Co on fiber digestibility, as well as cobalt balance (as determined by comparing the amount consumed to the amount lost via urine and feces). Further, this study compared varying amounts of dietary Co to serum Co and cobalamin concentrations. The hypothesis was that increasing dietary Co increases fiber digestion, as well as serum Co and cobalamin concentrations. 

## 2. Materials and Methods

### 2.1. Animal Care and Handling

Housing: Four mature Standardbred geldings (n = 4, mean body weight [BW] 503 ± 37 kg) were studied. Horses were housed at the Michigan State University Horse Teaching and Research Center in 3.7 × 3.7 m box stalls bedded with recycled and shredded newspaper. The Michigan State University Institutional Animal Care and Use Committee approved all methods (# 01/14-011-00). Weather permitting, horses were turned out daily in a fenced paddock. To prevent potential confounding factors caused by consumption of feedstuffs other than the provided diet, horses were not permitted turnout once the snow cover melted. During times when turnout was unavailable, horses were exercised at the walk on a mechanical walker for 30 min each morning, except during total collection periods.

Feeding: All horses were provided with ad libitum access to water and were initially fed a grass (mixed species) hay at 1.6% BW to meet projected baseline requirements, divided into two equal feedings per day. After each feeding, hay rations were assessed for feed refusal (hay left over from the previous feeding). Prior to the study, adequate quantities of a single hay source were sampled and then sent to Equi-Analytical Laboratories (Ithaca, NY, USA), where wet chemistry analysis was used to determine mineral concentration (Table 1). Hay samples were analyzed periodically throughout the duration of the study to ensure that the mineral intake remained consistent with the initial hay source analyzed. Only a single batch of hay was used to ensure nutrient profile consistency throughout the study. Further, no additional concentrates were provided, in an attempt to keep Co intake while on the control diet close to recommended amounts of 0.05 ppm.

Body Weight and Condition: Each week, horses were assessed for body condition score (BCS) [13] by two researchers and weighed on a digital scale to detect changes in body mass. Over the course of the study, forage intake was increased for two horses to maintain weight and body condition. 

Cobalt Supplementation: The 20-week study followed a 4 × 4 Latin square design divided into four 5-week periods. The first 2 weeks of each period were “washout” phases, during which the horses consumed only hay and were not provided a Co supplement. Though a Latin square design has the potential for a “carryover effect”, the initial 2 weeks of each period in which no Co was provided were designed to eliminate such. The washout period was then followed by a 3-week treatment phase. Each of the four horses was randomly assigned to one of four daily cobalt-supplementation treatments: 0.0 mg (control), 5.6 mg (low), 16.8 mg (medium), or 28.0 mg (high). The treatments were administered in tablet form via apple-flavored sucrose reward tablets produced by Purina TestDiet, which consisted of either “blank” tablets without Co or “cobalt” tablets formulated to contain 2.0% COPRO^®^25 (Zinpro Corporation, Eden Prairie, MN, USA) and deliver Co in the form of Co glucoheptonate (0.1 g COPRO/5 g tablet). At each of the two daily feedings, horses were provided 5 tablets consisting of the following number of each tablet to provide the required daily dosage of supplemental Co when fed twice per day: control (5 “blanks”, 0 “cobalts”); low (4 “blanks”, 1 “cobalt”); medium (2 “blanks”, 3 “cobalts”); high (0 “blanks”, 5 “cobalts”). With horses receiving 10 tablets daily, and with each tablet containing 4.7 g sucrose, the 10 tablets provided an additional 47 g of sucrose to each horse’s daily diet.

The prescribed treatment tablets were placed into feed buckets and were readily consumed by the horses. Table 2 shows the concentration of Co supplemented for each treatment. The treatment dosages were designed to result in the control diet providing Co near the current recommendation of 0.05 ppm, with increasing dosages that could be consistently provided by varying amounts of each of the treatment tablets. 

Collections: Total collections of urine and feces were performed on days 11 to 14 and days 32 to 35 of each period. Feces and urine were collected via a total collection harness (Equisan Marketing Pty Ltd., Melbourne, Australia). Each horse was fitted with a padded harness worn only for the duration of each collection. The harnesses were thoroughly washed with hot water and bleach after each use and were properly stored between collections. The harness remained with the respective horse for the remainder of the collections. Sampling took place for 3 d and excrement was collected every 8 h. When samples were collected, harnesses were emptied of all excrement; urine was transferred into clean vessels and feces were placed into a plastic bag. Both urine and feces were well mixed to ensure that the sub-sample collected was an accurate representation of the entire collection. Ten percent of each collection was retained and frozen for further analysis. Blood samples were drawn via jugular venipuncture with a 20 G needle and vacuum clot tube (BD Vacutainer^®^, Becton Dickenson, Franklin Lakes, NJ, USA), approximately two hours after the horses received their morning tablets on days 14 and 35 of each period, and preserved for serum analysis.

### 2.2. Sample Analysis

Hay Samples: Hay samples collected on days 11–14 and 32–35 of each period were ground in a Wiley Mill (Parr Instrument Company Inc., Moline, IL, USA) using a 1 mm screen, and stored in sterile bags for later analysis. Although researchers observed each horse for feed refusals during the collection periods, there were none from any of the subjects. 

Fecal and Urine Samples: At the end of each 72 h total collection, two 0.5 kg sub-samples of the composites of the nine fecal samples from each horse were retained and frozen at −20 °C. Subsequently, one fecal sub-sample per horse from each collection was freeze-dried. Similarly, the nine urine samples from each horse were combined to create a composite sample; two 100 mL sub-samples from each were retained. One sub-sample remained unaltered, while the second was treated with 2 μL of 12 M HCl acid to dissolve urine sediment. Each of these latter samples was further acidified with varying amounts of additional 12 M HCl to achieve a pH of 4 (Vitros Chemistry, Version 5). The dilution factor for each sample was calculated and accounted for accordingly. Acidified urine samples were submitted to Michigan State University Veterinary Diagnostic Laboratory (MSU VDL) to be analyzed via ICP mass spectroscopy for mineral concentration. 

Dry Matter (DM): Each sample of feces (freeze-dried and unaltered) and feed was evaluated for DM content. The unaltered feces were thawed at room temperature. Approximately 1 g of each fecal and feed sample and 0.5 g of freeze-dried feces were weighed and oven-dried at 105 °C for 24 h. The samples were weighed again to determine DM content. All DM samples were performed in triplicate with accepted CVs under 3%. 

Fiber Analysis: To determine acid-detergent fiber (ADF), neutral detergent fiber (NDF), and acid-detergent lignin (ADL), 0.5 g subsamples were analyzed using the procedures described by Van Soest et al. [14].

Microwave Digestion: Each of the ground, freeze-dried fecal and feed samples was prepared for microwave digestion. All vessels ran in the microwave digester at 180 °C, 1200 W, 200 PSI, for 30 min. After the vessels reached room temperature, 2.0 mL of 30% H_2_O_2_ was added to each sample.

Cobalt Analysis: Urine and microwaved digested samples were analyzed for Co content via inductively coupled plasma mass spectrometry (ICP-MS) by MSU VDL. Values were used to calculate daily Co intake, as well as average daily Co balance as a percentage of intake calculated by subtracting daily urinary and fecal mineral excretion from daily intake and dividing that by the amount consumed and multiplying by 100%.

Serum Evaluation: Blood samples from days 14 and 35 of each period were spun in a centrifuge at 3500× *g* for 20 min. Three aliquots of blood serum from each sample were collected and frozen at −20 °C. The frozen serum samples were analyzed by MSU VDL via ICP mass spectroscopy for Co concentration. Duplicate serum samples were sent to be analyzed for cobalamin and folate concentrations at the Gastrointestinal Laboratory in the Department of Small Animal Clinical Sciences at Texas A&M University. The samples were analyzed using the Immulite 2000 system (Siemens AG, Munich, Germany).

Statistical Analysis: Data from all measured variables were analyzed using SAS 9.2 (SAS Institute Inc., Cary, NC, USA). Variables BW, BCS, DMI, Co intake, Co balance, and serum Co concentrations were analyzed using repeated measures of the MIXED procedure with a Tukey–Kramer adjustment. Analysis of treatment groups had fixed parameters of horse, treatment, and period, while analysis of washout groups used only a fixed effect of period. Lignin, NDF, ADF, and DM digestibility were analyzed using repeated measures of the GLM procedure. Serum concentrations of cobalamin and folate were analyzed using repeated measures of the GLM procedure. All results are reported as LSM ± SEM. 

## 3. Results

### 3.1. Body Weight and Condition 

There were no treatment differences in BCS or BW (*p* = 0.9 and 0.08, respectively). Mean BCS during the study was 5.0 ± 0.9, while mean BW was 503 ± 36 kg. One horse developed a mild case of urticaria (“hives”) shortly after ending the high treatment, which became more prominent after the horse was on the control diet for a week. 

### 3.2. Dry Matter Intake, DM, and Fiber Digestibility

There were no treatment differences in DMI, which averaged 9.3 kg/day (*p* = 0.3), nor in DM, NDF, ADF, or lignin digestibility (Table 3). 

### 3.3. Cobalt Intake and Balance

Cobalt intake varied, corresponding with the varying amount of administered Co supplement. As expected, Co intake was lowest with the control treatment and highest with the high treatment (*p* < 0.0001; Table 4). Likewise, fecal and urinary Co were lowest for the control and low diets and highest for the high diets (*p* < 0.01). Co balance (fecal and urinary losses subtracted from the amount consumed) as a percentage of intake did not differ between the low (19%), medium (20%), and high (15%) treatments, but was lowest for the control treatment (−34%, SEM = 13%; *p* = 0.04). In comparison, the excretion as a percentage of intake ranged from 80% of the cobalt that was consumed being excreted in the medium group to 135% of the amount being consumed in the control group. 

Notably, peak urinary Co concentration was also highest for the high diet, being 34 ng/mL (*p* < 0.01; Table 5).

### 3.4. Serum Cobalt, Cobalamin, and Folate

**Figure 1:**
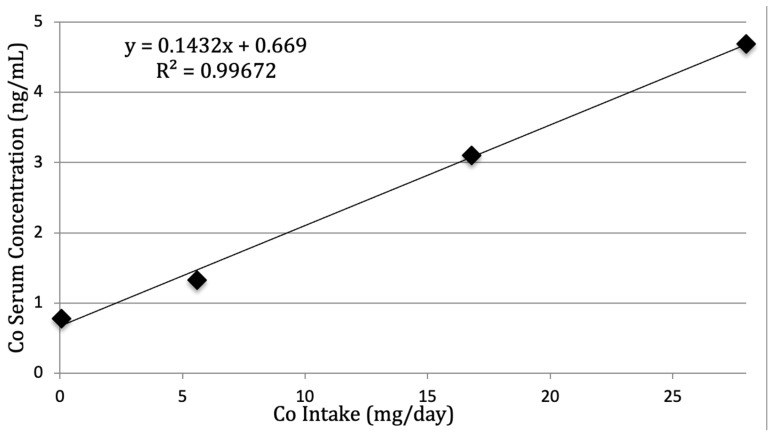
Serum Co concentrations (ng/mL) for each treatment measured on d 35 of each period.

Serum Co concentrations varied by treatment, with the lowest being while horses were on the control and low diets and the highest on the high diet (*p* < 0.001; Table 6). As shown in Figure 1, a linear regression of the intake values of Co used in the current study, against the serum Co concentrations, resulted in an equation: Y = 0.1423 (x) + 0.669, where Y = Serum Co Concentration and x = Dietary Co Intake. 

There was an observed treatment difference in serum concentrations of cobalamin (*p* = 0.003; Table 6). The highest serum cobalamin concentration was seen in the control group (2684 ± 25 pg/mL), while the lowest was seen in the high treatment group (2526 ± 25 pg/mL). While this difference is significant, it represents only a 6% difference. There was a lack of treatment differences in concentrations of serum folate, ranging from 8.9 ± 0.3 ng/mL on the control diet to 8.2 ± 0.3 ng/mL on the high diet (*p* = 0.09). 

## 4. Discussion

### 4.1. Fiber Digestion

Alterations in Co intake did not result in detectable differences in DM, ADF, NDF, or lignin digestibility. Prior preliminary research [15] led to the hypothesis that an increase in dietary Co had the potential to increase the microbial population of the equine hindgut, which is responsible for breakdown of indigestible fibers. Thus, it was expected that increasing Co would result in a higher fiber digestibility of consumed roughage. Recently, work in calves documented that increasing dietary Co increased both rumen cellulolytic microbial growth and fiber digestion [16]. Though higher fiber digestibility was not observed in the current study, addition of Co might have altered microbial population of the hindgut, but this was not assessed. In a 48-h in vitro study, addition of Co also failed to enhance fiber digestion using cecal fluid from horses, also suggesting that any increase in fiber digestion with Co supplementation is likely limited at best [6]. It should be noted that the negative lignin digestibility suggests that horses consumed some bedding (shredded newspaper). Thus, while the comparison in digestibility between treatments remains valid, it is likely that the calculated fiber digestibility values are slightly skewed due to an unknown quantity of bedding being consumed.

### 4.2. Cobalt Intake and Balance

As designed, there were differences in Co intake as treatments contained varying amounts of Co. The percentage of Co that was not excreted did not differ among the three Co-supplemented treatments, but was higher (*p* < 0.004) when compared to the control treatment. It should be noted that intake of Co on the control diet (0.06 ppm) slightly exceeded the recommendation provided by the 2007 Horse NRC (0.05 ppm) [17], which had been decreased from the 0.1 ppm recommendation by the 1989 Horse NRC [18]. When looking at the Co balance, more Co was being excreted than consumed when horses were on the control diet. That seems to suggest that endogenous losses are exceeding intake [17], and the recommendation of 0.05 ppm may not actually be meeting the requirements of the horses. However, in the control group, the amount being lost in the feces and urine beyond what was consumed only averaged 0.21 mg (which may effectively not be different from 0). By contrast, in the high group, of the 28.9 mg of Co consumed, 24.5 mg was excreted in the feces, and 1.4 mg was excreted in the urine, leaving a positive balance of 4.26 mg. Unless an animal is pregnant, lactating, or growing, or has a need to deposit minerals in other tissues (such as hair or hooves that are eventually lost and replaced), it would be surprising if minerals such as Co continued to accumulate in the body. It is reasonable to expect that if studies continue for an extended period of time, absorption rates might decrease such that losses match intake [17].

### 4.3. Serum Cobalamin (Vitamin B_12_) and Folate Concentrations

Serum cobalamin concentration did not increase with increasing amounts of dietary Co. These results differ from a 1966 study in which Co supplements were fed to pregnant sheep with apparent Co deficiency. In that study, Co supplementation increased serum vitamin B_12_ concentrations of ewes and their lambs [19]. In another ovine study, Co-deficient lambs had lower serum vitamin B_12_ concentrations than their healthy counterparts. After supplementation with Co, treatment groups showed higher B_12_ serum concentrations than did non-supplemented sheep [20]. Studies in cattle have yielded similar findings. Kincaid and Socha reported that dietary Co supplementation increased B_12_ concentrations in milk and colostrum of cows; however, Co concentrations in liver tissue or serum were unchanged [21]. With the primary site of the microbial population in the gastrointestinal tract differing between ruminants (foregut) and horses (hindgut), direct comparisons should be viewed with caution. Less work has been performed in horses, though Stillions et al. found a linear relationship between increases in B_12_ following increases in dietary Co [22].

The lack of an expected increase in serum cobalamin with Co supplementation may be due to insufficient time on each diet, or cobalamin concentrations may have been adequate without supplementation. Alternatively, there may have been an interaction with another nutrient in the diet that influenced results in this study. Another possibility is that when feeding Co in excess (in the low, medium, and high treatment groups), the response is not as great as when it is marginal or possibly slightly deficient (in the control group). Regardless, the difference between groups was only 6% and is likely not physiologically significant.

B_12_ cobalamin vitamins, together with folic acid, are critical for mitochondrial function [23], and it was hypothesized that Co supplementation might alter concentrations of folate. However, no changes were detected.

### 4.4. Serum Cobalt, Testing Thresholds, and Misuse

While few of the parameters measured had differences associated with varying amounts of dietary Co provided, the most useful information from this study pertains to serum Co concentrations. Despite feeding 60 times the 2007 Horse NRC [17] recommended amount of Co, serum Co concentration of the high treatment group was only 4.7 ± 0.3 ng/mL, as compared to 0.8 ± 0.3 ng/mL for the non-supplemented control diet. 

To discourage use of supplemental Co for potential performance-enhancing benefits, and to protect horse welfare, many racing jurisdictions in the United States have established a serum threshold of 25 ng/mL [24]. This value is also currently the international threshold established by the International Federation of Horseracing Authorities [25] as published in Section 16 of Article 6A of the International Agreement on Breeding, Racing and Wagering. Further, an international threshold of 100 ng/mL of Co in urine has also been established. Utilizing 245 Standardbreds with Co only provided through their diet, McKeever et al. reported a mean plasma Co of 1.03 ng/mL with a standard deviation of 0.92 ng/mL. These authors suggested raising the threshold to 71 ng/mL, as it would represent only a 1-in-10,000 risk of a false positive [24]. Notably, the horses in the current study had blood drawn about two hours after being fed the supplemental Co. When Kwak et al. [26] gave a single oral dose with alfalfa hay and measured urinary Co concentrations over a 48-day period, the highest concentrations were seen within 10 h, and the time for it to drop below threshold levels was 34 h. Given that horses in the current study received their dose of Co every 12 h, this suggests that detected amounts would likely be fairly consistent compared to what might be observed with only a single dose, as likely done when Co is administered to enhance performance.

When the Indiana Horse Racing Commission (IHRC) began conducting post-race blood tests for Co, they found serum concentrations as high as 353 ng/mL in some Standardbreds. One Thoroughbred tested after winning a race had a serum Co concentration of 1127 ng/mL—nearly 45 times higher than the threshold [11]. Using the equation featured in Figure 1, when serum Co concentrations meet the threshold set by the IHRC (25 ng/mL), the estimated dietary intake would be at 170 mg/day. Most troublesome, however, is that when serum levels reach 1127 ng/mL, the estimated dietary intake would be at 7900 mg/day. This is over 280 times the amount provided to the high treatment group in this study. Granted, there is no evidence that the relationship is linear when Co administration is beyond what was provided in this study. However, it does suggest that the amount provided to the racehorses testing positive was far beyond what one would feed in normal circumstances.

### 4.5. Influence of Cobalt Source

The most commonly used pharmaceutical drugs and oral preparations for Co supplementation provide, at the most, about 5 mg of Co daily (Table 5). According to a 2014 study evaluating commercial Co products, when the recommended dosage of the oral Co supplement “Hematinic” was administered, consequent urinary Co levels were as high as 113 ng/mL [9]. The subjects in our study received Co supplementation up to 28 mg of Co daily, with the highest observed urinary Co concentration being 34 ng/mL, well below the international threshold of 100 ng/mL. Granted, it is unknown whether sustained, long-term consumption of Co may produce a different response [26]. The same question exists for plasma Co, though Wenzel et al. reported an initial rise with weekly injections followed by an unexpected decrease by day 42 of their study [27].

Interestingly, the horses in this study were provided with up to five times the amount of daily Co as horses in the study evaluating Hematinic. However, administration of Hematinic yields peak urine concentrations nearly 3.5-fold of those found in this study. The data from such similar studies yield contradicting results; both studies provided Co orally, both measured urinary Co using IPC-MS, and still, the results are quite different. To ensure that the ingredients and concentrations listed on the label were indeed accurate, the researchers of our study chose to analyze the content of the Co tablets independently of the manufacturer. The MSU VDL found no discrepancies between the reported Co concentration by Zinpro Performance Minerals (the source of the Copro 25 used in this study) and the actual contents of the tablets. Therefore, it is appropriate to consider the possibility that the concentration in Hematinic may differ from that reported on the label, or that the Co source may have a different availability. This could hold major implications for the horse industry. Given equine welfare concerns, and also legal issues pertaining to performance testing thresholds, it is critical that consumers, veterinarians, and horse owners be aware of what is contained in the supplements they choose to provide their equine athletes. 

### 4.6. Limited Health Concerns

Side effects of high Co consumption have been shown to include thyroid dysfunction, myocardial necrosis, GI illnesses, nervous system failure, and sensory deprivation [28]. Further, intravenous administration of CoCl_2_ of up to 4 mg/kg weekly for 5 weeks induced hypertension and cardiac arrhythmias in Standardbreds [29]. Those studies could provide an explanation for a recent upsurge in the unexplained deaths amongst racehorses, which have been linked to an overload of supplemental Co. 

While the horses on the high treatment in this study consumed quantities 60 times greater than what is recommended by the 2007 Horse NRC [17], subjects showed no obvious adverse effects. However, this amount is nearly 90% less than the maximum tolerable concentration of 25 mg Co/kg DM as set by the 2005 NRC for other livestock species [30]. As noted previously, one of the horses developed a mild case of urticaria (“hives”). While likely not related to treatment, anecdotal skin reactions to cobalt have been noted [31]. Though the urticaria in the research horse cannot be definitively linked to Co, it is mentioned here in case future work with Co reports similar findings.

McKeever et al. administered 50 mg of elemental Co as CoCl_2_ intravenously to seven healthy Standardbreds on three consecutive days to investigate potential performance enhancement [24]. Serum Co concentrations increased from 1.6 ng/mL pre-administration to 369 ng/mL following three doses. The investigators failed to detect changes in performance indexes. Further, no adverse effects, even at this relatively high dose, were observed. Whether long-term parenteral administration of Co at high doses could result in a different response and health concerns is unknown. 

## 5. Conclusions

Supplementation with Co did not have any effect on DF digestion as hypothesized. Increasing Co intake did not increase serum vitamin B_12_ concentrations as predicted, though all treatments met the 2007 Horse NRC recommendations for Co. However, Co excretion through feces and urine slightly exceeded intake, resulting in a negative Co balance while on the control diet. This suggests that endogenous losses exceeded intake and may provide evidence that the 2007 Horse NRC recommendations are slightly low. A somewhat surprising finding was that serum cobalamin was higher under the control diet than under the high treatment, though the difference was minor and likely of little physiological importance.

As hypothesized, increasing dietary Co did increase serum Co concentrations. These results provide important information for the equine feed industry and consumers. With the widely publicized concerns of Co being misused in the racehorse industry, and the potential for health concerns and failed drug tests when Co is provided in large dosages, consumers have been wary of having any supplemental Co in prepared feeds. With many commercial feeds incorporating vitamin and mineral premixes to avoid dietary deficiencies, and with many of those premixes containing Co, this has caused challenges for the feed industry. This current study, along with the work by McKeever et al. (2020), provides reassurance that elevated amounts of dietary Co will not result in serum Co concentrations that could result in health concerns or approach thresholds established by racing jurisdictions. This provides support for the hypothesis that samples testing positive for elevated serum Co concentration likely result from administration of injectable solutions, rather than dietary Co in fortified feeds. 

## Figures and Tables

**Table 1:** Analysis of forage composition measured prior to the start of the study, analyzed by Equi-Analytical Laboratories, Ithaca, New York.

Unit Measured	As Fed	Dry Matter
% Moisture	13.1	
% Dry Matter	86.9	
% Ca	0.56	0.65
% P	0.29	0.34
% Mg	0.24	0.27
% K	2.24	2.58
% Na	0.015	0.017
% S	0.20	0.23
ppm Cu	6.5	7.4
ppm Zn	17.4	20.0
ppm Mn	59.6	68.6
ppm Mo	4.1	4.7
ppm Co	0.07	0.08

**Table 2:** Amount and concentration of Co supplement provided with each treatment, resulting in 1.2× (control), 14× (low), 40× (medium), or 60× (high) the recommended amount of dietary Co.

Treatment	Supplemental Dietary Co Provided (mg Co/day)	Total Dietary Co Including from Hay) on a mg/kg DM Basis
Control	0.0	0.06
Low	5.6	0.7
Medium	16.8	2.0
High	28.0	3.0

**Table 3:** Average digestibility of DM, ADF, NDF, and lignin during treatment periods, reported by treatment group, represented by the amount of supplemental Co added to their diet (control: 0 mg; low: 5.6 mg; medium: 16.8 mg; high: 28.0 mg).

	0	5.6	16.8	28.0	SEM	*p* Value
DM%	52	55	52	53	0.9	0.2
ADF%	49	50	46	48	1.3	0.3
NDF%	51	54	51	52	1.4	0.5
Lignin%	4	0.7	−5	−2	3	0.3

**Table 4:** Average daily intake of cobalt (obtained both from hay and from dietary treatment), fecal Co, urinary Co, and Co balance as a % of intake, reported by treatment group, represented by the amount of supplemental Co added to their diet (control: 0 mg; low: 5.6 mg; medium: 16.8 mg; high: 28.0 mg).

	0 mg	5.6 mg	16.8 mg	28.0 mg	SEM	*p* Value
Intake Co (mg)	0.6 ^a^	6.3 ^b^	17.6 ^c^	28.9 ^d^	1.4	<0.0001
Fecal Co (mg)	0.8 ^a^	5.1 ^a^	13.9 ^b^	24.5 ^c^	1.8	<0.0001
Urinary Co (mg)	0.008 ^a^	0.03 ^a^	0.08 ^ab^	0.14 ^b^	0.02	0.003
Co balance as % of intake	−35% ^a^	19% ^b^	20% ^b^	15% ^b^	13%	0.04

^abcd^ Means with different superscripts are different.

**Table 5:** Current pharmaceuticals and oral preparations for Co supplementation, their ingredients, and recommended dosages [9], as well as for the cobalt used in the current study.

Supplement	Cobalt Ingredient as Listed	Recommended Daily Dose	Co Equivalent per Daily Dose (mg)	Peak Urinary Co (ng/mL) Observed
Injections				
Hemo-15	Cyanocobalamin 150 μg/mL	10 mL	0.99	81–530
VAM^®^ Injection	Cobalt gluconate 0.7 mg/mL	11 mL	1.08	374–424
Oral				
Farrier’s Formula	Cobalt carbonate 1.9 mg/170 g	1 cup (255 g)	1.41	Not observed
Hemopar	Cyanocobalamin 800 μg/L Co sulphate monohydrate 9 mg/L	60 mL	0.19	Not observed
Hemantinic	Cyanocobalamin 180 mg/L Cobalt carbonate 110 mg/L	80 mL	4.99	56–113
Current Study				
CoPro^®^	Cobalt glucoheptonate; 2.5% Co	10 tablets	28	34

**Table 6:** Average serum cobalt (ng/mL) and cobalamin (pg/mL) concentrations measured in horses, reported by treatment group, represented by the amount of supplemental Co added to their diet (control: 0 mg; low: 5.6 mg; medium: 16.8 mg; high: 28.0 mg).

	0 mg	5.6 mg	16.8 mg	29.0 mg	SEM	*p* Value
Cobalt	0.8 ^a^	1.3 ^a^	3.1 ^b^	4.7 ^c^	0.3	<0.0001
Cobalamin	2684 ^c^	2612 ^b^	2591 ^b^	2526 ^a^	25	=0.003

^abc^ Means with different superscripts are different.

## Data Availability

Requests for data and further inquiries can be directed to the corresponding author (B.D.N).
